# Nitrites in Meat Products in Serbia: Harmful or Safe?

**DOI:** 10.3390/foods14030489

**Published:** 2025-02-03

**Authors:** Jasna Kureljušić, Jelena Maletić, Slavoljub Stanojević, Branislav Kureljušić, Jelena Petković, Ana Vasić, Tanja Bijelić

**Affiliations:** 1 Scientific Institute of Veterinary Medicine of Serbia, Janisa Janulisa 14, 11000 Belgrade, Serbia; jasnakureljusic@yahoo.com (J.K.); jelena.maletic@nivs.rs (J.M.); branislavkureljusic@yahoo.com (B.K.); tanjica90@gmail.com (T.B.); 2 Directorate for National Reference Laboratories, Batajnički drum 7, deo br. 10, 11186 Belgrade, Serbia; slavoljub.stanojevic@minpolj.gov.rs; 3 Veterinary Specialist Institute “Jagodina”, Boška Jovića 6, 35000 Jagodina, Serbia; jelena.petkovic@vsijagodina.com

**Keywords:** sodium nitrite, meat products, Serbia, public health concern

## Abstract

Nitrate and nitrite, commonly added to meat products as sodium or potassium salts, serve multiple functions such as developing characteristic flavor, inhibiting microbial growth, and controlling rancidity by preventing lipid oxidation. Nitrites are recognized for their potential health risks to humans. The present research aimed to determine the amount of nitrite in four meat product categories in Serbia over a period from 2015 to 2021. A total of 923 samples were analyzed, including 293 finely chopped sausages, 203 coarsely chopped sausages, 160 canned meats, and 267 smoked meats. The smoked meat category consisted of eight distinct products, such as smoked pork ribs, sirloin, and chicken drumsticks. An ISO 2918 method was used to measure the nitrite content. The average nitrite content, expressed as sodium nitrite (NaNO_2_), was found to be 61.5 mg/kg in finely chopped sausages, 57.6 mg/kg in coarsely chopped sausages, 48.4 mg/kg in canned meat, and 41.8 mg/kg in smoked meat. The results collectively demonstrate nitrite concentrations within regulatory limits. In conclusion, the nitrite concentrations in all tested products were below the maximum allowable limits as per national and European regulations, ensuring compliance with safety standards while highlighting the importance of continuous monitoring to mitigate public health risks.

## 1. Introduction

The preservation of meat through the addition of salt followed by drying and smoking is a traditional practice widely adopted in Mediterranean countries and the Balkans. This method results in what are commonly referred to as cured products. Historically, unpurified salt contained nitrate, which enhanced its preservative action. The use of nitrate and nitrite in meat preservation dates back centuries, often implemented without a scientific understanding of their effects [1]. Research on the role of saltpeter (potassium nitrate) as a preservative for cured meat began in the late 1800s. In the early twentieth century, studies conducted in the United States and Europe significantly advanced the understanding of nitrite and nitrate’s importance in meat products. By the 1950s and 1960s, these compounds became standard ingredients in the meat industry [2]. Regulatory frameworks for nitrate and nitrite usage began to emerge in various countries in the 1920s; however, it was during the 1970s that such regulations became more consistent, albeit with notable differences between nations. [3].

Nitrate (NO_3_^−^) and nitrite (NO_2_^−^) salts play a vital role in the preservation of meat products, contributing significantly to their flavor, color, and safety. These additives are commonly employed to inhibit bacterial spoilage and prevent lipid oxidation, which helps control rancidity and enhances the sensory qualities of smoked and cured meats. The incorporation of nitrates and nitrites not only improves the quality and shelf life of meat products but also positions these compounds at the forefront of discussions about sustainable food systems, climate change, and healthy eating. Research indicates that these compounds can form carcinogenic N-nitroso compounds, raising questions about their long-term effects on human health [4,5,6]. Concerns about the potential health risks associated with high levels of nitrate and nitrite consumption persist. Ongoing scrutiny of their health implications underscores the importance of balanced consumption and regulatory oversight in the food industry [7].

The European Commission classifies nitrites (potassium nitrite, E249; sodium nitrite, E250) and nitrates (sodium nitrate, E251; potassium nitrate, E252) as permitted food additives under Commission Regulation (EU) No. 1129/2011 [8]. Notably, nitrites are associated with the formation of methemoglobin in the blood, which impairs oxygen transport. In 2015, the International Agency for Research on Cancer (IARC) classified ingested nitrates and nitrites as “probably carcinogenic to humans,” particularly under conditions that promote endogenous nitrosation [9]. While the established nitrite levels in cured meats are deemed safe, excessive and prolonged consumption can pose health risks [10].

The addition of nitrite anion (NO_2_^−^) stabilizes the red color by being reduced to nitric oxide (NO), which acts as a ligand that binds to both myoglobin and metmyoglobin. Upon heating, nitrosylhemochrome species are formed from the dark-red NO complexes, resulting in the characteristic pink-red color of cooked cured meat [11]. In cured meats, the distribution of nitrite occurs among various components, including protein (20–30%); myoglobin (5–15%); lipid (1–5%); and other species, such as nitrate (1–10%) [12].

For long-shelf-life meat products, the simultaneous addition of both nitrate and nitrite is crucial to ensure a continuous source of nitrite over extended periods. According to Commission Regulation (EU) Nos. 1129/2011 and 1333/2008 [13], the nitrite anion must originate from permitted food additives, which must be clearly labeled. Other sources of nitrites include fermented vegetable extracts rich in nitrates, such as carrot (171 µg/g), celery (2114 µg/g), beet (2273 µg/g), and spinach (3227 µg/g) [12]. Notably, commercial celery juice powder has been found to contain up to 27,462 µg/g (approximately 2.75%), indicating significantly higher concentrations post-drying [14]. Often, meat products made with these vegetable extracts are labeled as “produced without nitrite,” which can mislead consumers. The EU regulation establishes the maximum amounts of additives that can be added to meat products but does not differentiate based on their source [15,16].

Analytical methods for monitoring nitrite levels in meat typically involve the detection of residual nitrite using molecular absorption spectrophotometry based on the Griess reaction or high-performance liquid chromatography (HPLC) [17]. Regulation (EC) No. 1333/2008 [18], Annex II, Part E, food category 8.3 ‘Meat products’ lays down maximum amounts for potassium nitrite (E 249) and sodium nitrite (E 250) that may be added during manufacturing. The maximum added amount is 150 mg/kg for meat products in general and 100 mg/kg for sterilized meat products. For a few specified cured meat products made traditionally in specific Member States, the maximum added amount is 180 mg/kg [19]. Recent discussions have highlighted the regulatory aspects regarding food additives, emphasizing the importance of correct application and interpretation of the regulations [18].

Meat products in Serbia are a fundamental part of the nutrition of inhabitants. The main products of the customer’s choice are sausages, ham, and prosciutto [20]. The maximum limits of nitrite intake for meat products according to the Rulebook of the Republic of Serbia, known as the Rulebook on Food Additives, Official Gazette of the RS No. 53/18, are defined at a value of 150 mg/kg [21]. The exception to the normal restrictions on curing ingredients is bacon, which must have an incoming level of 120 mg/kg nitrite for immersion cured and 200 mg/kg nitrite for fiddled bacon. According to the Rulebook on the quality of minced meat, semi-finished meat, and meat products, Official Gazette of the Republic of Serbia, number 50/19 [22], the group of finely chopped sausages include hot dogs, frankfurters, Parisian sausages, and white sausages; the group of coarsely chopped sausages includes Serbian sausage, Tyrolean sausage, and mortadella; the group of canned meat in pieces includes cooked ham, cooked shoulder, cooked sirloin, cooked chicken fillet, and cooked bacon; and the group of smoked meat products includes ham, shoulder, sirloin, beef, poultry fillet, and drumstick.

The meat industry in Serbia is subject to legal regulations, so it is very important from that perspective to comply with the prescribed allowable limits of additives. Considering the harmful effects of nitrites as additives in meat products, this study aimed to determine their content in meat products available on the market in the Republic of Serbia.

## 2. Materials and Methods

### 2.1. Sampling

The samples were collected from the territory of the Republic of Serbia in the period from 2015 to 2021. Meat products (n = 923) were produced in eight factories of the meat industry over the course of seven years. A total of four meat products, namely, finely ground sausage, coarsely ground sausage, canned meat in chunks, and smoked meat were used in this study. The number of samples tested per meat product and year of sampling is provided in Table 1.

Smoked meat (n = 267) consisted of smoked chicken drumstick (n = 5), smoked pork neck (n = 42), smoked pork meat in pieces (n = 16), smoked sirloin steak (n = 62), smoked pork cheek (n = 8), smoked bacon (n = 88), smoked pork ribs (n = 19), and smoked pork hocks (n = 27). The total number of tested samples of smoked meat is provided in Table 2. 

The samples, in the amount of 0.5 kg per sample, were packed in plastic bags. Following the cold chain, they were delivered to the laboratory and stored at a temperature of 2 °C to 8 °C in a refrigerator until the beginning of the analysis. Before the analysis, they were taken out of the refrigerator and homogenized, and then they were taken for work and analysis not further than 48 h after sampling.

### 2.2. Method and Sample Preparation

ISO 2918 [23] outlines a standardized method to accurately measure compounds using spectrophotometry, which is applicable across various food matrices, including meats, vegetables, and beverages. The core principle of ISO 2918 is based on the reduction of nitrate to nitrite, followed by a colorimetric reaction. The food samples were homogenized thoroughly to ensure uniformity by passing them twice through a meat grinder and mixer (Waring Commercial, Stamford, CT, USA). A total of 10 g of samples was weighed and quantitatively transferred to an 300 mL Erlenmeyer flask. Then, 100 mL hot water, at a minimum temperature of 70 °C, along with a 5 mL saturated borax solution, were added sequentially to the flask. The sample was heated for 15 min in a boiling water bath while being shaken multiple times to ensure proper extraction. After cooling to room temperature, reagents were added: 2 mL potassium hexacyanoferrate (II) trihydrate and 2 mL zinc acetate dihydrate, with careful stirring after each addition. The sample was then transferred to a 200 mL container, made up to the mark with water, and mixed. It was allowed to settle for 30 min at room temperature. Following this, the top layer was decanted and filtered through folded filter paper to obtain a clear solution. An aliquot of the filtrate was pipetted and transferred to a 100 mL vessel. Water was added to achieve a final volume of approximately 60 mL before the addition of 10 mL reagent I (sulfanilamide and hydrochloric acid) and 6 mL reagent III (hydrochloric acid). The mixture was stirred and left for 5 min at room temperature in the dark. Subsequently, 2 mL reagent II (N-naphthyl-1-ethylenediamine chloride) was added and mixed in, and the mixture was allowed to sit for 3 to 10 min in the dark before being made up to the line with water.

The absorbance was measured on the spectrophotometer (Helios Gamma spectrophotometer (Thermo Scientific, Waltham, MA, USA)) using a 1 cm optical path cuvette.

### 2.3. Calibration Line

Calibrators were prepared by diluting various working standard sodium nitrite solutions in water and diluting them to about 60 mL final volume. The concentrations of sodium nitrite in the solutions were 2.5 μg/mL, 5.0 μg/mL, and 10.0 μg/mL [23].

### 2.4. Statistical Analysis

The XLSTAT for the Microsoft Excel (v. 2023) program was used for statistical analyses. To select the appropriate statistical test for analyzing the obtained results, a series of normality tests were applied to test the normality of the distribution of measured nitrite concentration, including the Anderson–Darling, Lilliefors, Shapiro–Wilk, and Jarque–Bera tests. The results were evaluated using a combination of descriptive statistical test with confidence interval (CI) of 95% and inferential statistical tests, such as Welch’s ANOVA, and post hoc tests (Tukey HSD, Games–Howell, and two-sided Dunnett). Confidence intervals were calculated with the Wilson score method. *p*-values < 0.05 were considered significant.

Homoscedasticity and independence of the error terms are key hypotheses in ANOVA where it is assumed that the error terms’ variances are independent, identically distributed, and normally distributed. The existence of heteroscedasticity is a major concern in analysis of variance, as it invalidates statistical tests of significance that assume that the modelling errors all have the same variance. To be sure whether it is necessary to correct heteroscedasticity and apply the Welch ANOVA test or not, a Durbin–Watson test was applied. The heteroscedasticity of residuals and variability in measured nitrate concentrations were evaluated across different product groups, particularly within the smoked meat group, which showed the most pronounced variations in nitrite concentrations.

## 3. Results

In the period from 2015 to 2021, the obtained results of nitrite concentrations in finely chopped cured sausages, coarsely chopped cured sausages, canned meat pieces, and smoked meat were as presented in Table 3.

These results collectively demonstrate a consistent commitment to maintaining nitrite concentrations within regulatory limits in smoked meat products throughout the assessed years. Despite the heterogeneity of measured nitrite values in different products, no increased values of nitrite concentration above the maximum permissible limits were registered.

**Figure 1:**
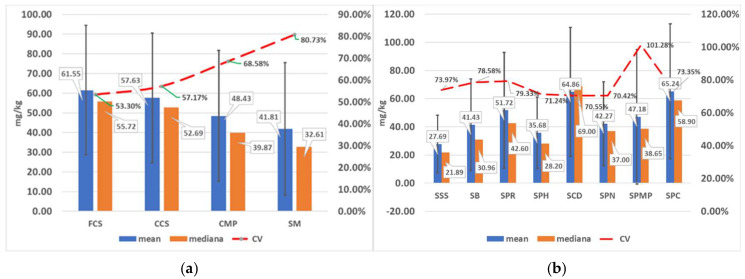
(**a**,**b**) Descriptive statistics of measured values of nitrite concentrations in different meat products and smoked meat products. FCS-finely chopped cured sausage; CCS-coarsely chopped cured sausage; CMP-canned meat in pieces; SM-smoked meat. SSS-smoked sirloin steak; SB-smoked bacon; SPR-smoked pork ribs; SPH-smoked pork hocks; SCD-smoked chicken drumstick; SPN-smoked pork neck; SPMP-smoked pork meat in pieces; SPC-smoked pork cheeks.

Figure 1a presents the descriptive statistics of the measured nitrite concentrations across different groups of meat products. The mean values, depending on the product group, ranged from 41.809 mg/kg in smoked meat to 61.546 mg/kg in finely chopped sausages, with 95% confidence intervals of 37.732 to 45.886 mg/kg and 57.766 to 65.327 mg/kg, respectively. The median values varied between 32.61 mg/kg in smoked meat and 55.72 mg/kg in finely chopped sausages.

Given that products from the smoked meat group showed the greatest heterogeneity in terms of varying nitrite concentrations, within this group of products, nitrite concentrations were analyzed separately by type of smoked meat. Results are presented in Figure 1b.

When comparing different groups of meat products, the observed differences in the measured concentrations of added nitrites were statistically significant (Welch statistic: F = 18.869, *p* < 0.0001; Brown–Forsythe F-ratio: F = 19.108, *p* < 0.0001), except in the case of finely chopped sausages and coarsely chopped sausages, where the nitrite concentrations between these two product groups did not differ significantly. The same applied to canned meat products in pieces and smoked meat. Table 4 and Table 5 show the results of the descriptive statistics and the assessment of the significance of differences in the measured nitrite concentrations between different product groups.

**Figure 2:**
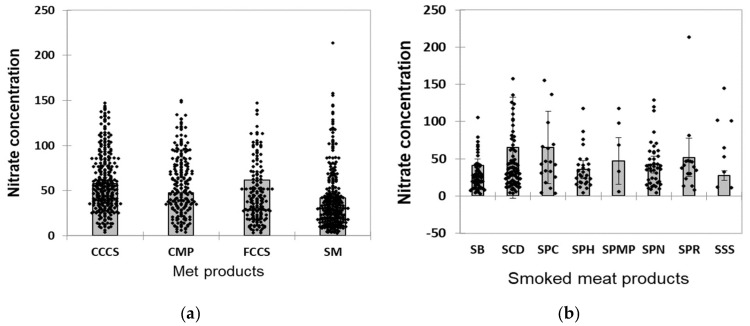
(**a**,**b**) Distribution of measured nitrite values in meat products and smoked meat products stratified by product groups. FCCS-finely chopped cured sausage; CCCS-coarsely chopped cured sausage; CMP-canned meat in pieces; SM-smoked meat. SSS-smoked sirloin steak; SB-smoked bacon; SPR-smoked pork ribs; SPH-smoked pork hocks; SCD-smoked chicken drumstick; SPN-smoked pork neck; SPMP-smoked pork meat in pieces; SPC-smoked pork cheeks.

Figure 2a shows the distribution of measured nitrite values in meat products stratified by product groups. It was necessary to observe a significant dispersion of the measured values from the central tendency, especially for products from the smoked meat group. In Figure 2b, the measured values show a statistically significant dispersion of the measured values, i.e., the coefficient of variation (CV) ranged between 53.30% and 80.58%. Regarding the between-group variability, the variance of different meat group products did not differ statistically between different products, except for the smoked meat category, i.e., F tests for heteroskedasticity were F = 0.637, *p* = 0.591, and F = 2.658, *p* = 0.011, respectively.

## 4. Discussion

Meat and meat product spoilage are influenced by several factors, including pH, water activity, and nutrient levels, which can promote the rapid proliferation of microorganisms during storage. To combat this, nitrites are frequently employed in the food industry as preservatives, effectively inhibiting the growth of spoilage microorganisms and food-borne pathogens, e.g., inhibition of *Clostridium botulinum* from producing botulinum toxin [24]. Sodium nitrite offers several benefits for curing meat, but controversy has surrounded it due to research suggesting that it may cause human cancer. There is a rising demand from customers for meat that is organic or has a low nitrite level. It is critical to lessen the quantity of nitrites added to meat products because of the detrimental effects nitrites have on human health. The use of plant extracts instead of synthetic nitrite seems to be appropriate [25].

Nitrite content was measured in 923 samples of meat products in Serbia. The representation of product groups in our complete population of analyzed products reflected their demand and consumption in Serbia. Among our product groups, the highest levels of consumption are products such as finely chopped sausages, coarsely chopped sausages, canned meat cuts, and smoked meat products. The highest values of nitrites were found in smoked meat, while in other products, the nitrite concentration is mostly below the limit of the maximum allowed concentration of 150 mg/kg.

In this study, the large variabilities of nitrite content for most meat products were observed. A major strength of this study is that in the nitrite concentration data, both mean and median values are presented, including results on maximum measured values and variability of detected values within different groups of meat products. There was apparent data skewness in all the meat product groups we analyzed (non-normal distribution; for more information, see Supplementary Material, Table S1 and Figures S1 and S2), which indicates the impossibility of predicting concentrations in these products; thus, we would propose using median nitrate concentrations rather than mean values when assessing associations between dietary nitrite intake and health outcomes, or calculating safe amounts of daily nitrite intake when preparing meals, especially when it comes to nutrition recommendations for sensitive categories of consumers [16]. In such situations, it may even be safer to opt for stricter criteria based on the use of the 95th percentile. When we analyzed the measured concentrations of nitrites by product groups, we found that their distribution was extremely heterogeneous. The results of the statistical analysis of nitrate concentration showed that the level of bound nitrates, which reflect the stability of nitrite concentrations during the technological process, depends on the type of products and the method of preparation. This process is difficult to predict, making regular control of finished products highly justified. Given that products from the smoked meat group showed the greatest heterogeneity in terms of varying nitrite concentrations, to determine how nitrite concentrations vary among different smoked meat products, we also analyzed these types of products separately. In the case of these products, although there was no significant variance difference between different types of smoked meat, the overall deviations of individually measured values from the mean value of nitrite concentration were even more pronounced (the coefficient of variation of nitrite concentrations ranged between 70.42% and 101.28%).

The residual levels of nitrite and nitrate in meat products remain a significant concern for consumer health, particularly due to their association with potential toxicological effects. This study revealed mean residual nitrite and nitrate levels ranging from 41.809 mg/kg in smoked meat to 61.546 mg/kg in finely chopped sausages, with median values between 32.61 mg/kg in smoked meat and 55.72 mg/kg in finely chopped sausages. These findings provide critical insights into the current state of nitrite and nitrate levels in processed meat products and highlight the need for stricter regulatory oversight.

When comparing these results to the maximum nitrite and nitrate levels reported in the literature, the values observed in this study are notably higher than those in sausages (4.6 mg/kg in South Korea and 139 mg/kg in Iran) and ham (7.7 mg/kg in Italy and 31.3 mg/kg in Korea). This discrepancy may be attributed to differences in manufacturing practices, preservation techniques, or regulatory enforcement between regions. Additionally, the observed higher residual levels in finely chopped sausages compared to smoked meat align with the expectation that products with finer structures and higher surface areas may retain more additives [26]. The European Union and its member states have implemented stricter regulations on the use of nitrites and nitrates, setting limits on their application in treatments and residue levels in meat products. Similarly, China, as the world’s largest meat producer, has introduced restrictions on nitrites and nitrates in cured meats, which are widely consumed traditional foods. Other countries, including Canada, Korea, and Japan, have also established limits on the use and residue levels of nitrites and nitrates in meat and meat products [26]. Our comparison of nitrite and nitrate levels between Jansen et al. (2018) [27] and our own findings highlights significant differences. We found that the samples analyzed in Jansen et al.’s study had considerably lower concentrations of both nitrites and nitrates. Specifically, the nitrite levels in corned beef and smoked beef were 7.4 to 47.4 mg/kg lower than those found in smoked meat and finely chopped sausages, while the nitrate levels were 9.2 to 55.8 mg/kg lower compared to the processed meats in our study.

The multifunctional properties of nitrates and nitrites make it challenging to find a single alternative that can fully replicate their roles in meat preservation. These compounds confer several benefits in cured meat products, including enhancing color, developing characteristic flavors, providing antimicrobial action, and serving as antioxidants. The formation of a nitrosyl myoglobin complex through the reaction of nitric oxide with myoglobin (deoxymyoglobin and metmyoglobin) is crucial for achieving the distinctive color associated with dried meats [28].

While the exact mechanisms by which nitrites and nitrates contribute to the unique aroma of cured meats are not entirely understood, research indicates that when nitrites interact with lipids and proteins, they yield various compounds. For instance, the binding of nitrites to sulfur-containing amino acids in meat proteins results in the formation of SH residues that impart specific aromas and flavors, enhancing the overall sensory experience of the dried product [29].

The antimicrobial properties of nitrite stem from its ability to inhibit bacterial metabolic enzymes, thereby limiting oxygen uptake and disrupting proton gradients. Notably, nitrite is effective against *Clostridium botulinum* spores. Additionally, nitrites and nitrates play a role in combating lipid oxidation by scavenging oxygen. Nitric oxide’s capacity to react with various radicals, including hydroxyl, alkoxy, and peroxyl radicals, allows it to interrupt radical chain reactions and bind to transition metals, further contributing to the stability and quality of cured meat products [30].

The thermal treatment of cured meat products significantly enhances used meat products reactivity, particularly due to the heat’s effect on nitrite ions, which become highly reactive in environments with a pH below 7 [31]. Nitrite ions interact with various meat components, including amino acids, sulfhydryl groups, amines, phenolic compounds, ascorbic acid, and myoglobin, and can act as nitrosating agents, leading to the formation of nitroso compounds [32]. Other nitrosating agents, such as nitrous acid and nitric oxide, also originate from nitrite. Nitrous acid contributes to the endogenous formation of N-nitroso compounds (NOCs), while nitric oxide plays a role in generating nitrates and nitrites that circulate within the human body. NOCs can be categorized into six types: non-volatile N-nitrosamines, volatile N-nitrosamines, N-nitrosated heterocyclic carboxylic products, N-nitrosamides, Amadori compounds, and N-nitrosated glycosylamines. Volatile nitrosamines, many of which belong to the 2B group of potentially carcinogenic substances, are particularly concerning [33]. The levels of nitrosamines in processed meats vary depending on the product type, with concentrations sometimes falling below detectable limits (one microgram per kilogram). These compounds are typically formed during high-temperature cooking or meat processing [34]. Epidemiological studies have suggested a potential link between nitrate, nitrite, and N-nitroso compounds and increased cancer risk, particularly about N-nitroso dimethylamine, which is considered a more potent carcinogen. However, despite these associations, there is currently no definitive evidence linking the consumption of processed meats to an elevated cancer risk, although high exposure to nitrites from various sources has been implicated in increased health risks [35].

Sodium nitrite’s role as a precursor to nitrosamines has raised public concern regarding its use in meat curing due to the potential health risks. While it is understood that there is a positive relationship between the amount of nitrite added and nitrosamine formation, this relationship is not linear. Nitrosamines are organ-specific, meaning that certain types only cause cancer in specific organs. They are also known to exhibit teratogenic effects, with approximately 97% of the 300 identified nitrosamines being teratogenic in animal studies [36]. Nitrosamines in meat are formed through a complex process influenced by various factors, including nitrite, nitrate, primary and secondary amines, amides, peptides, proteins, and amino acids, which can be converted into nitrosamine precursors by microbial activity [37]. Nitrosamines can develop during meat production or home cooking, or within the digestive tract after consumption. In cured meats, residual nitrite may react with amines and free amino acids under conditions such as the presence of secondary amines, low pH, temperatures above 130 °C, and the availability of NO2 [38]. The formation of nitrosamines, particularly during grilling or frying, occurs in small amounts but is considered a potential cancer risk even with prolonged exposure to low levels [39]. Despite this, the concentrations of these compounds in cured meats are generally low, but the concern arises from the possibility of chronic exposure leading to carcinogenic effects over time [10].

## 5. Conclusions

Nitrite is an essential additive in meat and meat products, as its multiple functions in meat processing cannot yet be fully replaced by other substances. To ensure the safe use of nitrite and its oxidized form, nitrate, various countries and regions have implemented regulations and guidelines. The study found that nitrite concentrations in all analyzed meat products were below the maximum allowable limits according to national and European regulations, ensuring their safety. However, continuous monitoring remains essential to mitigate potential public health risks associated with nitrite consumption.

To meet evolving consumer demands and safety standards, the meat industry is actively exploring alternatives that can replace nitrite’s multifaceted functions. Plant extracts, organic acids (like lactate and sorbate), and high-pressure processing (HPP) are among the potential substitutes being investigated. Nevertheless, further research is essential to develop cost-effective and efficient solutions that ensure the safety, quality, and flavor of meat products while minimizing health risks associated with nitrite.

## Figures and Tables

**Table 1:** Number of samples per meat product category and year of sampling.

Year/Meat Product	Finely Ground Sausage (No.)	Coarsely Ground Sausage (No.)	Canned Meat in Chunks (No.)	Smoked Meat (No.)
2015	39	51	18	45
2016	44	40	13	38
2017	47	20	37	42
2018	31	15	23	19
2019	35	10	14	23
2020	49	23	21	41
2021	48	44	34	59
Total	293	203	160	267
Total sum: 923				

**Table 2:** Results of measured nitrite concentrations in meat product categories stratified by years of production (2015–2021).

Meat Products			Finely Chopped Cured Sausage (Results ± SD)	Coarsely Chopped Cured Sausages (Results ± SD)	Canned Meat in Pieces (Results ± SD)	Smoked Meat (Results ± SD)
Nitrite concentration in mg/kg	2015	No. of samples	39	51	18	45
		Average value	80.6 ± 30.4	77.0 ± 37.2	74.5 ± 39.4	44.3 ± 31.3
	2016	No. of samples	44	40	13	38
		Average value	83.3 ± 32.7	61.8 ± 30.1	68.8 ± 31.9	59.8 ± 39.5
	2017	No. of samples	47	20	37	42
		Average value	91.4 ± 26.0	71.6 ± 24.3	62.9 ± 35.1	63.0 ± 44.2
	2018	No. of samples	31	15	23	19
		Average value	57.6 ± 24.5	60.8 ± 39.9	55.0 ± 33.5	34.0 ± 25.9
	2019	No. of samples	35	10	14	23
		Average value	35.9 ± 22.1	33.4 ± 19.4	29.7 ± 14.7	24.9 ± 14.8
	2020	No. of samples	49	23	21	41
		Average value	40.3 ± 17.1	40.7 ± 20.1	28.6 ± 13.2	24.4 ± 15.5
	2021	No. of samples	48	44	35	59
		Average value	39.9 ± 13.4	38.3 ± 19.4	27.3 ± 13.5	34.4 ± 26.6

**Table 3:** Analysis of the differences between the meat product categories (nitrite concentration): Games–Howell post hoc test. (significant results are in red, non-significant results are presented in green color in the table).

Contrast	Difference	Standardized Difference	Critical Value	*p*-Value	Significant	Lower Bound (CI = 95%)	Upper Bound (CI = 95%)	Lower Bound (CI = 95%)	Upper Bound (CI = 95%)
SM vs. FCCS	−19.737	−7.005	2.577	<0.0001	Yes	−27.038	−12.437	▌▌▌	
SM vs. CCCS	−16.088	−5.193	2.579	<0.0001	Yes	−24.130	−8.046	▌▌▌▌	
SM vs. CMP	−6.616	−1.980	2.582	0.197	No	−15.311	2.078	▌▌▌▌	▌
CMP vs. FCCS	−13.121	−4.036	2.582	0.000	Yes	−21.583	−4.660	▌▌▌▌▌	
CMP vs. CCCS	−9.472	−2.709	2.582	0.036	Yes	−18.576	−0.368	▌▌▌▌▌▌	
CCCS vs. FCCS	−3.649	−1.216	2.579	0.617	No	−11.438	4.139	▌▌▌	▌

FCCS—finely chopped cured sausage; CCCS—coarsely chopped cured sausage; CMP—canned meat in pieces; SM—smoked meat. Statistically significant results are in red, statistically non-significant results are presented in green color in the table).

**Table 4:** Measured nitrite concentrations across different categories of meat products.

Group of Meat Products	Mean Nitrate Conc. (mg/kg)	Standard Error	Lower Bound (95%)	Upper Bound (95%)	Summary of All Pairwise Comparisons (Games—Howell)	
FCCS	61.546	1.926	57.766	65.327		B
CCCS	57.897	2.326	53.333	62.462		B
CMP	48.425	2.65	43.224	53.627	A	
SM	41.809	2.077	37.732	45.886	A	

FCCS—finely chopped cured sausage; CCCS—coarsely chopped cured sausage; CMP—canned meat in pieces; SM—smoked meat. Gradation of colors represents the concentration mean values from high in red to low in green.

**Table 5:** Measured nitrite concentrations across different groups of smoked meat.

Meat Product	Mean Nitrate Conc. (mg/kg)	Standard Error	Lower Bound (95%)	Upper Bound (95%)	Summary of All Pairwise Comparisons (Games–Howell)	
SPC	65.238	2.666	22.444	32.942		B
SCD	64.860	5.123	26.734	46.911		B
SPR	51.723	3.530	34.475	48.378		B
SPMP	47.181	4.761	32.890	51.642		B
SPN	42.266	13.161	21.265	73.097		B
SB	41.426	10.208	31.622	71.825		B
SPH	36.823	28.600	8.542	121.178	A	
SSS	27.693	20.669	24.537	105.938	A	

SSS—smoked sirloin steak; SB—smoked bacon; SPR—smoked pork ribs; SPH—smoked pork hocks; SCD—smoked chicken drumstick; SPN—smoked pork neck; SPMP—smoked pork meat in pieces; SPC—smoked pork cheeks. Gradation of colors represents the concentration mean values from high in red to low in green.

## Data Availability

The original contributions presented in this study are included in the article/Supplementary Material. Further inquiries can be directed to the corresponding author.

## Supplementary Materials

The following supporting information can be downloaded at: https://www.mdpi.com/article/10.3390/foods14030489/s1, Table S1. The result of testing the normality of the distribution of measured nitrite concentrations in meat products. Figure S1. The result of testing the normality of the distribution of measured nitrite concentrations in meat products: PP and QQ plot of measured nitrate concentration. Table S2. ANOVA statistics of measured values of nitrite concentrations in different meat products. Figure S2. Analysis of the variability of the measured nitrite concentrations and the normality of the residual distribution of the measured and predicted values. Figure S3. Mean value and 95 CI of nitrite concentrations in meat products stratified by group of products.
