# Supplementary material for: Nitrites in Meat Products in Serbia: Harmful or Safe?
Source: Foods. 2025 Feb 3;14(3):489. doi: 10.3390/foods14030489 (PMC11816862; doi:10.3390/foods14030489)
Supplement: Supplementary file 1 [file foods-14-00489-s001.zip › foods-3424745-supplementary.pdf]

Table S1. The result of testing the normality of the distribution of measured nitrite concentrations in meat products

Shapiro-Wilk test:		Anderson-Darling test:		Lilliefors test:		Jarque-Bera test:	
W	0.9591	A2	3.3178	D	0.0827	JB (Observed value)	19.3353
p-value	< 0.0001	p-value	< 0.0001	D (standardised)	1.4161	JB (Critical value)	5.9915
alpha	0.05	alpha	0.05	p-value	< 0.0001	DF	2
				alpha	0.05	p-value	< 0.0001
						alpha	0.05

Test interpretation:

Ha: The variable from which the sample was extracted does not follow a Normal distribution.

As the computed p-value is lower than the significance level alpha=0.05, one should reject the null hypothesis H0, and accept the alternative hypothesis Ha.

The risk of rejecting the null hypothesis is H_0 while it is true is lower than 0.01%.

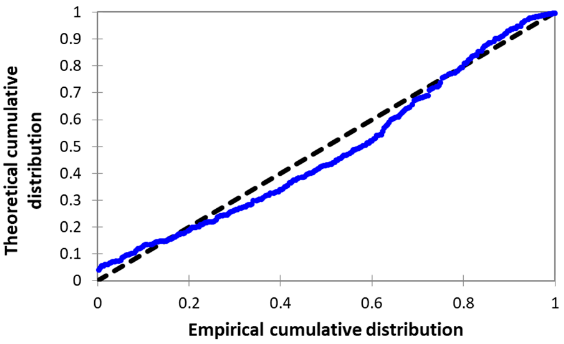

Fig. S1a. PP plot of measured nitrate concentration in meat products

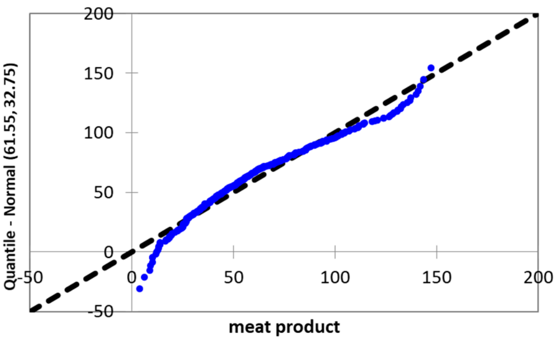

Fig. S2a. QQ plot of measured nitrate concentration in meat products

Figure S1. The result of testing the normality of the distribution of measured nitrite concentrations in meat products: PP and QQ plot of measured nitrate concentration

Table S2. ANOVA statistics of measured values of nitrite concentrations in different meat products (FCCS - finely chopped cured sausage; CCCS-coarsely chopped cured sausages; CMP-canned meat in pieces; SM-smoked meat)

Analysis of variance:					
Source	DF	Sum of squares	Mean squares	F	Pr > F
Model	3	62457.2071	20819.0690	18.9063	< 0.0001
Error	919	1011974.9540	1101.1697		
Corrected Total	922	1074432.1611			

Computed against model Y=Mean(Y)

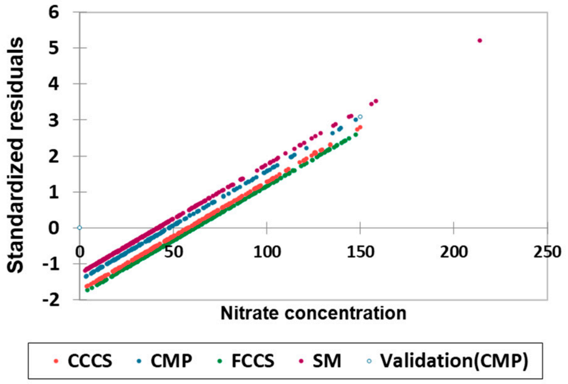

Fig. S2a. Nitrate concentration/Standardized residuals

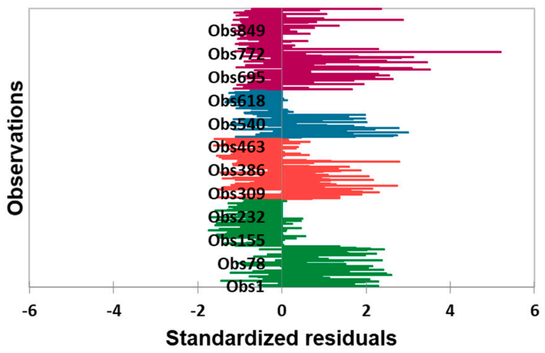

Fig. S2b. Standardized residuals/Nitrate concentration

Figure S2. Analysis of the variability of the measured nitrite concentrations and the normality of the residual distribution of the measured and predicted values

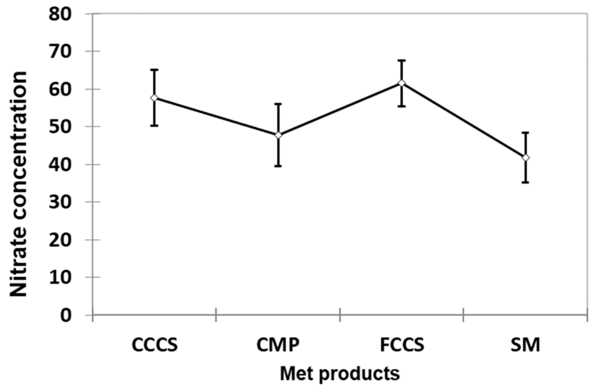

Fig. 3a. Mean nitrite concentrations in different meat products

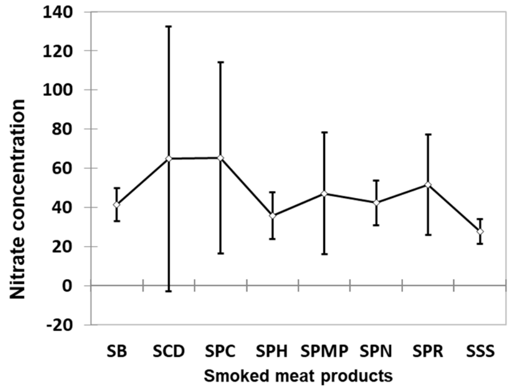

Fig. 3b. Mean nitrate concentrations in different smoked meat products

Figure S3. Mean value and 95 CI of nitrite concentrations in meat products stratified by group of products
